# EHMTI-0354. Abnormal expression of gene transcripts linked to inflammatory response in the periosteum of chronic migraine patients: implications to extracranial origin of headache

**DOI:** 10.1186/1129-2377-15-S1-K2

**Published:** 2014-09-18

**Authors:** R Burstein, C Perry, P Blake, C Buettner, M Bhasin

**Affiliations:** 1 Anesthesia, Harvard Medical School, Boston, USA; 2 Surgery, Memorial Hermann Northwest Hospital, Houston, USA; 3 Neurology, Memorial Hermann Northwest Hospital, Houston, USA; 4 Medicine, Harvard Medical School, Boston, USA; 5 Genomics Proteomics and Bioinformatics, Harvard Medical School, Boston, USA

## Introduction

Progress in headache research suggests that migraine pathophysiology involves inherited abnormal brain functions and that the initiation of headache depends on flow of nociceptive signals that originate in pain-sensitive intra- and extra-cranial organs conveyed through peripheral nociceptors to central trigeminovascular neurons. Despite this progress, little is known about the identity of the pain fibers that mediate the initiation phase, or what activates them.

## Aims

To assess the extent of presence of inflammatory molecules in periosteum tissues that were taken from calvarial areas where the head hurts.

## Methods

We compared number of copies of gene transcripts (mRNA) that encode proteins that play known roles in inflammatory and immune responses and number of copies of molecules that regulate the expression of those genes (miRNA) in periosteum tissues of 27 chronic migraine (CM) patients and 17 control subjects.

## Results

36/524 mRNAs and 27/726 miRNAs were differentially expressed in the periosteum of CM patients compared to control subjects. Of these, 25 genes were up-regulated (all encode proteins that facilitate inflammation) whereas 11 were down-regulated (all encode proteins that suppress inflammation). Of the 27 post-transcripts, expression was low for 11 (all regulate expression of pro-inflammatory genes) and high for 16 (all regulate expression of anti-inflammatory genes) miRNA sequences.

## Conclusion

The findings suggest that the molecular environment in which periosteal pain fibers exist contain abnormal expression of genes that promote inflammation; a condition that is likely to lower their activation threshold, when symptoms appear at the onset of an attack, or activate them chronically.

